# Clay Aerogel Supported Palladium Nanoparticles as Catalysts

**DOI:** 10.3390/gels2020015

**Published:** 2016-04-08

**Authors:** Jared J. Griebel, Matthew D. Gawryla, Henry W. Milliman, David A. Schiraldi

**Affiliations:** Department of Macromolecular Science and Engineering, Case Western Reserve University, Cleveland, OH 44106-7202, USA; jgriebbs@gmail.com (J.J.G.); mgawryla@hotmail.com (M.D.G.); henry.milliman@gmail.com (H.W.M.)

**Keywords:** aerogel, catalyst, palladium

## Abstract

Highly porous, low density palladium nanoparticle/clay aerogel materials have been produced and demonstrated to possess significant catalytic activity for olefin hydrogenation and isomerization reactions at low/ambient pressures. This technology opens up a new route for the production of catalytic materials.

## 1. Introduction

Owing to their high surface area to volume ratios and associated optimal utilization of precious metals required to carry out catalytic transformations, supported nanoscale metal particles are finding great interest as catalysts for organic synthesis [1,2,3,4], as well as in electrodes for proton exchange membrane fuel cells [5,6]. Mesoporous silica and amorphous carbon are typical supports for metal nanoparticles, due to their large surface areas and relative abundance and low cost. Lower density versions of these supports, such as silica aerogels and carbon aerogels may further extend the range of useful supported metal nanoparticle catalysts. Silica aerogels possess many qualities that are useful in supported catalysts such as low density, high surface area, and high thermal stability [7]. Carbon aerogels have found significant use when catalysts are utilized in electronic applications [8]. Clay aerogels, an inexpensive alternative to silica and carbon aerogels, are produced via an environmentally friendly freeze drying process [9]. Clay aerogels have already been demonstrated to be useful in producing rigid [10], elastomeric [11], electrically conductive [12] and temperature responsive polymer composites [13], and given their high ion-exchange capacities could be imagined to be useful in a wide range of other applications. The ease of use of bio-based polymers as binders for the clay skeletons, and the inherently low flammability of these materials without the addition of any flame retardants render these materials to be sustainable [14,15,16,17]. Because of their inherent ability to be converted into ceramic materials, clay-based aerogels can be processed into desired macroscopic geometries, then thermally treated to produced durable, porous ceramic catalysts with inherently low pressure drops, high surface areas, and relatively high metal loadings.

We report the use of a metal nanoparticle/clay aerogel as a heterogeneous catalyst herein. We are only familiar with one other reference in this area [18]. These catalysts were produced by preparing nanoscale palladium particles, supported on sodium montmorillonite clay (Na-MMT) followed by conversion of the metal/clay into aerogel structures. Both the Pd/clay and Pd/clay aerogels were demonstrated to be useful for the isomerization and hydrogenation of 1-octene.

## 2. Results and Discussion

### 2.1. Catalyst Preparation and Characterization

**Figure 1:**
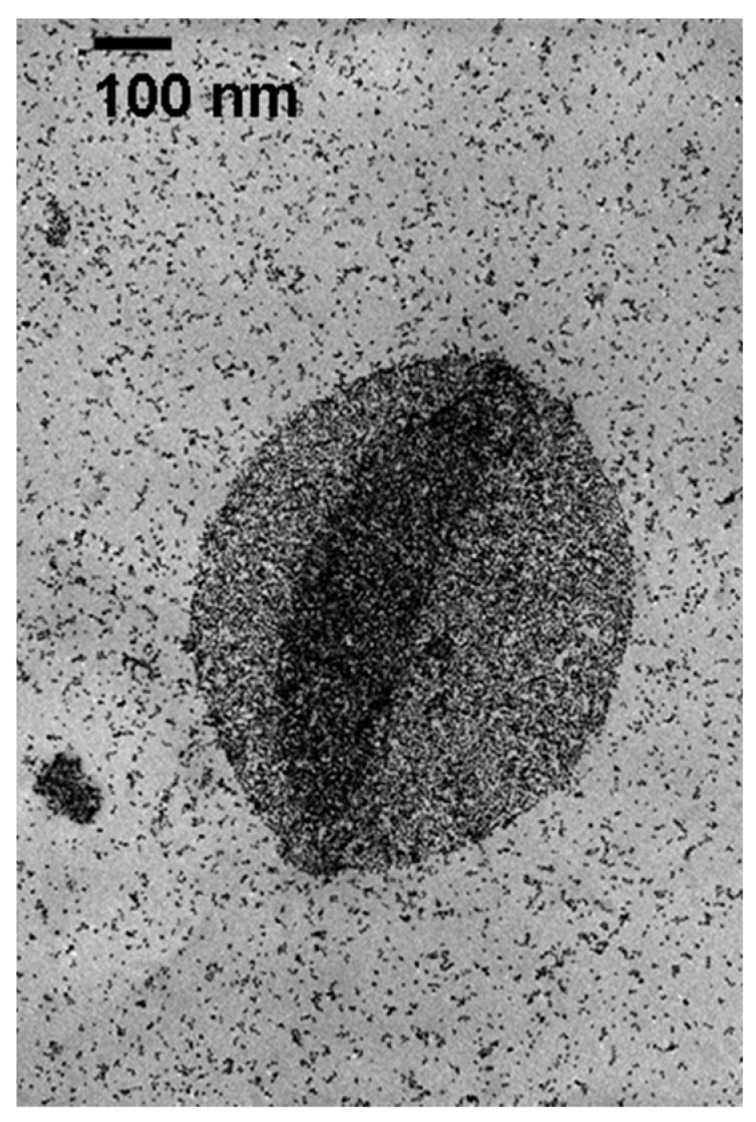
TEM image of Pd-MMT.

Clay-supported palladium nanoparticles (Pd-MMT) were produced from palladium(II) in the presence of sodium montmorillonite, with ethylene glycol as the reducing agent using a procedure adapted from other workers, but used by our group to produce Pt/C catalysts for fuel cell applications [6]. The clay/metal combination prepared in this manner, Pd-MMT, was then converted into aerogels via freeze drying, using a variation of our published procedure [9]. Ethylene glycol reduction of the palladium acetate solutions yielded a visible color change, qualitatively indicating the formation of nanoparticles. The solution color changed from a mustard yellow at the combination of the Pd(II)Ac/MMT/dioxane mixture and ethylene glycol to a dark grey color after four hours of reduction. TEM images were taken of the resulting clay supported palladium nanoparticles, showing a distribution of particles in the 4–6 nm range were formed, along with occasional large aggregates of NPs (Figure 1). The presence of aggregates in such metal nanoparticle materials has been observed by a number of workers; variations in synthesis temperature, concentration, reducing agent, and supporting surfactant have been shown to affect the particle size and size distributions [5,6].

**Figure 2:**
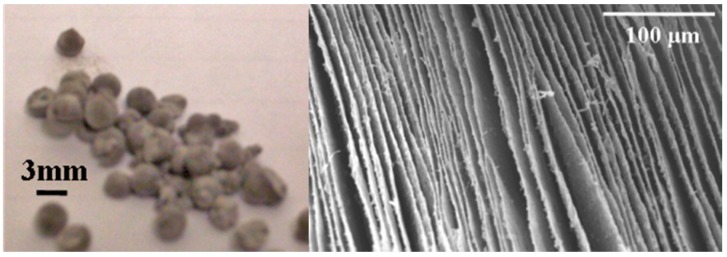
Pd-MMT aerogel bead structure (**left**) Internal structure of beads (**right**).

**Figure 3:**
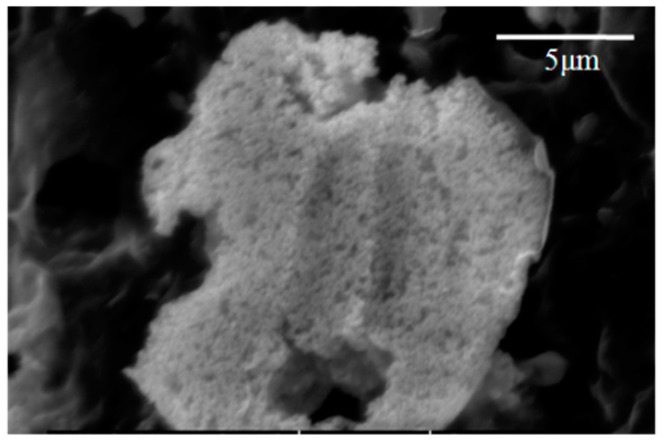
Cluster of Pd-MMT.

While stable gels of montmorillonite can be formed at 2 wt % in water with pristine clay, the aerogels made from such a low solids level are extremely fragile and will not withstand extended handling [9]. In order to produce a material that is sufficiently robust for normal use, clay levels of at least 5 wt % were utilized in the initial hydrogels which were to be freeze dried. Initial trials showed that the Pd-MMT clay did not form stable gels in solution; it was necessary to mix modified and unmodified clay to produce a stable wet gel. Clay gels are stabilized by ionic interactions in between clay layers; the presence of palladium nanoparticles appear to mask this charge and prevent a stable gel from forming. A stable gel incorporating the Pd-MMT clay was produced from the combination of 2.5 wt % Pd-MMT clay with 3.75 wt % pristine clay. Figure 2 (left) shows that the aerogel beads, before use as catalysts, were approximately 3–5 mm in diameter and spherical to hemispherical in shape; an SEM image (right) of the layers within the bead is given and shows the lamellar structure typical of such freeze dried aerogels [9,10]. These beads were produced by dropwise addition of aqueous clay gel into liquid nitrogen. As the water froze into ice, a layered structure was generated; expansion during the freezing process typically cracked the beads into two hemispheres. SEM images show clusters of Pd-NPs (Figure 3) distributed over the surfaces of the aerogel. While these clusters may be the primary form of Pd in the material they appear highly porous, composed of the 4–6 nm primary particles in a “popcorn ball” type of structure, and are evenly decorated across the entire aerogel support.

Samples were characterized by SEM both prior to and after the hydrogenation reaction. It was observed that during the hydrogenation reaction, mechanical action of the magnetic stirring bar used to mix reactants caused the aerogel particles to deteriorate slightly.

BET Surface analysis of the nanoparticle/MMT/aerogel was carried out using nitrogen as the test gas; a value of 43 m^2^/g was determined, which is in the 30–60 m^2^/g range that is typical for polymer/clay aerogels produced via freeze drying, in our experience. The palladium therefore did not substantially change this surface area characteristic.

### 2.2. Catalytic Reactions of the Pd/MMT Aerogel

The conditions for the isomerization and hydrogenation reactions were adapted from those of Liu [19]. Isomerization reactions were run under atmospheric pressure/temperature in a three-neck round bottom flask using a mixture of 5% hydrogen 95% nitrogen (nitrogen diluent used for safety reasons). As this is a preliminary study, only one loading of palladium was examined. Hydrogenation reactions were run in a Fisher-Porter pressure vessel at a constant 3.8 atm pressure; operated as a sealed, batch system, only initial and final samples were taken for analysis. The octene to catalyst mol ratios of 100–300:1 were employed for these studies, with hexanes used as dilutents for their low boiling point, allowing easy product separation.

**Figure 4:**
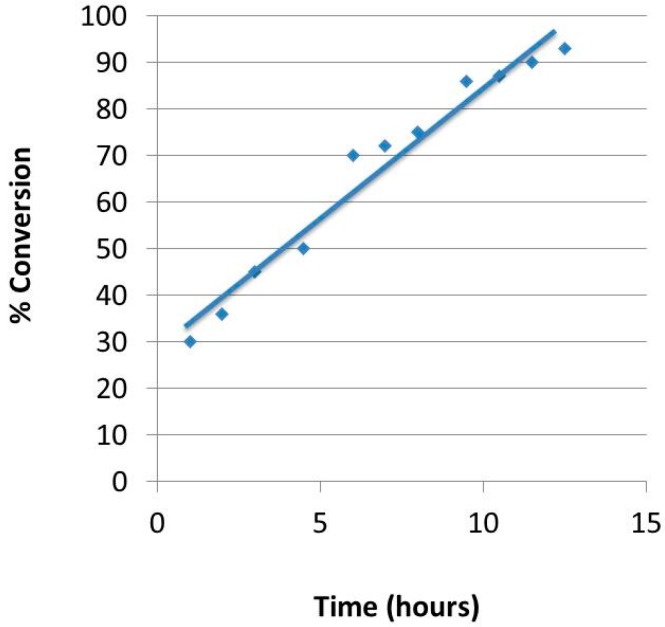
Conversion of 1-octene to other isomers.

**Figure 5:**
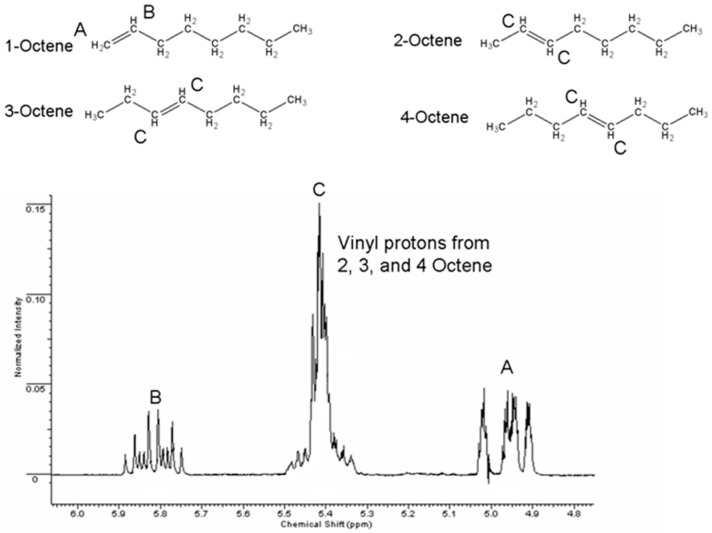
NMR spectra containing the integrations of the two vinyl protons from 1-octene and the vinyl protons from the other isomers.

Operating at atmospheric pressure and under mixed gas (0.05 atm hydrogen partial pressure) no hydrogenation of 1-octene was observed. Isomerization of the alpha olefin to 2-, 3- and 4-octenes was observed instead. Such activation of C=C bonds is well known [20] and indicates that catalytically-active palladium surfaces are available after processing of the clays. Such a palladium-catalyzed isomerization of 1-octene has been previously reported [21]. With the low hydrogen partial pressure used in this preliminary study, hydrogenation would be unexpected. The layered structure of the Pd-NP/clay catalyst was maintained during use as a catalyst, although some attrition to fine particles was observed in the magnetically-stirred, slurry reaction system employed in the present study. Operation of a continuously fed, packed bed hydrogenation reactor should minimize such attrition. The conversion of 1-octene to products was quantified by comparison of the vinyl protons in the ^1^H NMR spectra of samples taken hourly; GC/MS analysis further supported the NMR values. Figure 4 shows the monotonic approach to thermodynamic equilibrium between the octene isomers as the starting 1-octene was isomerized. The percentage of trans-2-octene to the cis isomer was calculated for each experiment by comparing the integral of the former’s methyl group found at 1.64 ppm to the latter’s methyl peaks at 1.58 and 1.60 (Figure 5); the percentage of trans to cis 2-octene in the reaction flask increased slightly from 52% to 60% of the total 2-octene presence over 12 h of reaction time. The conversion from 2-octene to 3-octene was monitored using the methyl protons next to the vinyl group on 2-octene compared to the total vinyl groups in the mixture. The average turn over number (TON) measured 10 moles of 1-octene converted to 2-octene per mole of Pd per hour for the non-aerogel material. When structured into an aerogel bead an average TON of 6 h^−1^ was calculated under the relatively low substrate:catalyst conditions employed.

Hydrogenation reactions were carried out a constant hydrogen pressure (3.8 atm). After the reaction had run for the desired time, the pressure was released, the contents were decanted to separate the catalyst from the solution and the solution was separated by distillation. Hexanes were used as an unreactive diluent to achieve the desired volume within the reaction flask, and then easily removed since the boiling point of hexanes is 69 °C while the boiling points of octene and octane are 122 and 125 °C, respectively. Once separated from the hexanes, proton NMR and GC/MS were used to determine the reaction products. A 47% conversion of 1-octene to octane was accomplished in 15 min; 97% conversion was obtained in 30 min, with quantitative production of octane observed after 1 h (Table 1). These hydrogenation conversions correspond to an initial turn over number of approximately 200 h^−1^ until such time that substrate is largely exhausted. It should be noted that similar TON values have been reported for palladium and platinum on silica/alumina [22,23] and chitosan [24] supports although most of the prior literature studies generally use much higher hydrogen pressures [25]; such catalysts can be useful in the production of jet aircraft fuel [26]. Because the isomerization turn over numbers for the aerogel and the clay/palladium powder were similar, hydrogenation with the power itself was not attempted.

As was noted above, the supported palladium catalyst is efficient at isomerizing the alfa olefin into its more thermodynamically stable isomers. This isomerization can be seen in the data presented in Table 2. A control experiment with palladium-free clay aerogel, in contrast, showed no detectable conversion of the starting 1-octene.

Combining the isomerization and hydrogenation results, one can propose a mechanism in which the starting alpha olefin is coordinated to palladium in the well-known π-allyl form, followed by reversible hydride transfer to the metal center and isomerization of the organic substrate [27]. In the presence of hydrogen gas, metal hydrides can be produced, leading to hydrogenation of the olefins to produce the octane product.

## 3. Conclusions

The synthesis and utility of a novel solid support for nanoparticle catalysts has been shown herein. Clay aerogels that have been decorated with palladium nanoparticles have been shown to be viable hydrogenation catalyst system. The high porosity of the aerogel combined with the reactivity of palladium should allow for their utilization in flow-through reactors. The possibility of converting these materials into ceramics is currently being investigated as a way to reinforce the structure and limit the attrition during stirred reactions.

## 4. Experimental Section

### 4.1. Materials

Palladium(II) acetate, hexanes, octane, 1-octene (Sigma-Aldrich, St. Louis, MO, USA); 1,4-dioxane, ethylene glycol, tetrahydrofuran(THF) (Fisher, Waltham, MA, USA); sodium montmorillonite clay (Na-MMT; PGW grade, Nanocor, Arlington Hts, IL, USA) were all used as received. Five percent Hydrogen/95% nitrogen gas mixture (Airgas, Radnor, PA, USA) was used for safety reasons rather than pure hydrogen.

### 4.2. Nanoparticle Preparation

Palladium nanoparticles (PdNPs) were produced using a procedure adapted from Ahmed [6,13]. Palladium(II) acetate (0.471 g) was dissolved in 25.0 mL of 1,4-dioxane in a round bottom flask using a magnetic stirrer. Na-MMT (0.500 g) was added to the flask and allowed to mix until a homogeneous dispersion was observed. Ethylene glycol (75.0 mL) was cooled in a separate 250 mL round bottom to 0 °C using an ice bath. Once the ethylene glycol had reached 0 °C, the Pd(II)Ac/PGW/dioxane mixture was added with continuous stirring and the temperature was raised to 150 °C. The entire system was fitted with a continuous argon purge prior to heating. The reaction was held at 150 °C for 4 h during which time the color of the mixture changed from brown to a dark grey. After the reaction was completed, it was removed from the heat and the clay was allowed to settle. The liquid was decanted and the clay was washed with tetrahydrofuran three times to remove any residual solvents or by-products. After the final washing, the material was allowed to air dry in a glass petri dish (theoretical Pd content = 31 wt %). The clay/metal combination synthesized via this method will be referred to as Pd-MMT.

### 4.3. Aerogel Preparation

Clay aerogels were prepared using a variation of our published procedure [9]. Clay/water gels containing 6.25 wt % solids were produced by shearing clay water mixtures on high (~22,000 rpm) in a Waring model MC-2 mini laboratory blender for 2–3 min. The 6.25% wt % solids was typically made up of 2.5 wt % Pd-MMT and 3.75 wt % pristine MMT clay (when only Pd-decorated clay was used, aerogels were not produced). The resulting gel suspensions appeared homogeneous, with no evidence of phase separation over the duration of their use, although it should be noted there were some larger particles suspended within the gel. This suspension was then dropped from a pipette into liquid nitrogen to form hemispherical particles approximately 3–5 mm in diameter. The frozen particles were then dried using a Virtis Advantage EL-85 freeze dryer (Warminster, PA, USA) where high vacuum (3 µbar/25 °C) was applied to sublime the ice and to produce aerogel structures.

### 4.4. Characterization

Scanning electron microscopy was performed using a Phillips XL-ESEM scanning electron microscope (Leuven, Belgium). Samples were observed both prior to and after the hydrogenation reaction. A Varian 300 MHz NMR (Palo Alto, CA, USA) was used to observe the progression of the reactions and final products. Transmission Electron Microscopy (TEM) images were taken of the Pd-MMT decorated clay prior to creating the aerogel. The images were collected on a JEOL 1200 EX TEM (Peabody, MA, USA) operating at an accelerating voltage of 80 kV. TEM samples were prepared by dispersing small amounts of material in methanol; a small drop of this solution was allowed to evaporate on the TEM grid. Gas Chromatography coupled with a mass spectrometer (GC/MS) was carried out on a HP 5890 Series II GC (Agilent Technologies, Santa Clara, CA, USA) with an HP 5971 Mass Selective Detector; a J&W Scientific DB-5MS (30 m, 0.25 mm ID, Agilent Technologies) non-polar column was used. Ultra high purity helium was used with a flow rate of 1 mL/min. The temperature program was held isothermal at 30 °C for 15 min with a solvent delay of 5 min (all analytes eluted between 5.5 and 12.5 min). A five point calibration curve was constructed for both octane and 1-octene (ranging from 1 to 50 ppm in hexanes) both having linear regressions of 0.996. The reaction products were diluted to a total concentration of 50 ppm in hexanes. All calibration standards and samples were run in triplicate and the averages used to determine the compositions.

BET Surface area was measured using nitrogen and a Micromeritics TriStar II Surface Area/Porosimeter (Norcross, GA, USA) using a 139 mg sample of aerogel/nanoparticle.

### 4.5. Safety Precaution

When trying to recover the palladium-decorated clay, vacuum filtering or evaporation of methanolic solutions results in spontaneous ignition. Tetrahydrofuran solutions did not ignite and, therefore, were used instead.

### 4.6. Catalytic Reactions—Isomerization

The conditions for the reactions were adapted from those of Liu [18]. Isomerization reactions were run under atmospheric pressure in a three-neck round bottom flask using a mixture of 5% hydrogen 95% nitrogen (nitrogen diluent used for safety reasons). Octene (20.0 mL) was combined with 0.38 g of Pd-MMT (not in aerogel form; MMT clay + PdMMT = 0.38 g) in a 50.0 mL round bottom flask, corresponding to a catalyst:substrate mole ratio of 120:1. The reaction was run for 12 h with 0.20 mL aliquots taken every hour for analysis.

### 4.7. Catalytic Reactions—Hydrogenation

Hydrogenation reactions were run in a 400 mL Fisher-Porter pressure vessel (Fisher, Waltham, MA, USA) at a constant 3.8 atm pressure (again, 5% hydrogen in nitrogen blend was used), fitted with a 60 psi relief valve, pressure gauge and hydrogen inlet. Operated as a sealed, batch system, only initial and final samples were taken for analysis. The typical reaction mixture consisted of 38.7 mL of hexanes solvent and 11.3 mL of 1-octene with 0.469 g of aerogel catalyst beads. The octene to catalyst mol ratio was 106:1, however the large size of the reaction vessel required a diluent for adequate stirring during reaction. Hexanes were used for their low boiling point, allowing easy product separation.

## Figures and Tables

**Table 1:** Hydrogenation of 1-octene catalyzed by palladium/aerogels.

Reaction Time (h)	Percentage of Total Composition		
	Unreacted 1-octene	2-, 3-, and 4-octene	octane
0.25	3	50	47
0.5	4	5	91
1	nd	Nd	100
12	nd	Nd	100

**Table 2:** Summary of Catalytic Reaction Conditions.

Sample	Amount of Pd-PGW (g)	Moles of Pd in Reaction	Moles of 1-Octene	Measured Conversion over 12 h	Mol Ratio of Substrate to Catalyst	12 h Avg. TON (h^−1^)
Isomerization Powder	0.38	1.1 × 10^−3^	0.13	97%	120	10
Isomerization Aerogel	0.0828	2.4 × 10^−4^	0.064	25%	270	6
Hydrogenation Aerogel	0.1876	5.5 × 10^−4^	0.072	100%	300	11 *

* This reaction is largely complete after 30 min; a more accurate TON value would be 200 h^−1^.
